# Functional role of autophagy in testicular and ovarian steroidogenesis

**DOI:** 10.3389/fcell.2024.1384047

**Published:** 2024-05-17

**Authors:** Ali Afzal, Yue Zhang, Hanan Afzal, Umair Ali Khan Saddozai, Lei Zhang, Xin-Ying Ji, Muhammad Babar Khawar

**Affiliations:** ^1^ Shenzhen Institute of Advanced Technology, University of Chinese Academy of Sciences, Shenzhen, Guangdong, China; ^2^ Molecular Medicine and Cancer Therapeutics Lab, Department of Zoology, Faculty of Science and Technology, University of Central Punjab, Lahore, Pakistan; ^3^ Department of Obstetrics and Gynecology, 988 Hospital of People's Liberation Army, Zhengzhou, Henan, China; ^4^ Institute of Translational Medicine, Medical College, Yangzhou University, Yangzhou, Jiangsu, China; ^5^ Henan International Joint Laboratory for Nuclear Protein Regulation, School of Basic Medical Sciences, Henan University, Kaifeng, Henan, China; ^6^ Faculty of Basic Medical Subjects, Shu-Qing Medical College of Zhengzhou, Zhengzhou, Henan, China; ^7^ Department of Medicine, Huaxian County People’s Hospital, Huaxian, Henan, China; ^8^ Applied Molecular Biology and Biomedicine Lab, Department of Zoology, University of Narowal, Narowal, Pakistan

**Keywords:** autophagy, testicular steroidogenesis, ovarian steroidogenesis, testosterone, progesterone

## Abstract

Autophagy is an evolutionarily conserved cellular recycling process that maintains cellular homeostasis. Despite extensive research in endocrine contexts, the role of autophagy in ovarian and testicular steroidogenesis remains elusive. The significant role of autophagy in testosterone production suggests potential treatments for conditions like oligospermia and azoospermia. Further, influence of autophagy in folliculogenesis, ovulation, and luteal development emphasizes its importance for improved fertility and reproductive health. Thus, investigating autophagy in gonadal cells is clinically significant. Understanding these processes could transform treatments for endocrine disorders, enhancing reproductive health and longevity. Herein, we provide the functional role of autophagy in testicular and ovarian steroidogenesis to date, highlighting its modulation in testicular steroidogenesis and its impact on hormone synthesis, follicle development, and fertility therapies.

## Introduction

Autophagy is referred to as an intracellular catabolic pathway that is genetically determined and evolutionarily conserved from yeast to higher primates (Vargas et al., 2023). In typical physiological contexts, autophagy mediates the internalization of cellular cargoes such as; old protein and damaged organelles with lysosomes thereby sequestering and reutilizing to maintain cellular homeostasis (Debnath et al., 2023). Lysosomal degradation further characterizes a common endpoint of various autophagic mechanisms, such as chaperone-mediated autophagy (CMA), microautophagy, and macroautophagy, hither ahead denoted as autophagy; each with distinct purpose and indiscriminate sequestration and degradation (Levine and Klionsky, 2004; Ma et al., 2023). Additionally, cells engage other specialized mechanisms for selective targeting, such as lipophagy, zymophagy, mitophagy, and crinophagy; to target specific substrates (Vargas et al., 2023). The former ones are usually referred to as “bulk” or “non-selective” autophagy and the latter ones are denoted as “selective” autophagy. While the existence of autophagy has been recognized for some time, its comprehensive exploration has only recently gained momentum. Besides its homeostatic functions, autophagy significantly influences the disease course of almost all cancers, various infections, immune responses, and multi-organ disorders, as well as neurodegenerative conditions (Nanayakkara et al., 2023; Yamamoto et al., 2023). Autophagy works as a quality-control process, removing invading pathogens, protein masses, and dysfunctional or senescent proteins and organelles from cells (Yao et al., 2021). Additionally, it plays important roles in diverse processes such as cellular differentiation, embryonic development, and aging, potentially offering protective effects (Wang et al., 2019; Aman et al., 2021). The recognition of autophagy’s substantial implications in various diseases has heightened research interest in exploring its physiological and pathological aspects.

Earlier, we have discussed autophagy in relation to lipolysis (Khawar et al., 2019; Khawar et al., 2021a) in the liver (Nazeer et al., 2023) and reproduction (Gao et al., 2019; Gao et al., 2020; Khawar et al., 2022). Interestingly, extensive research on autophagy in normative and pathological endocrine settings has yielded promising knowledge, whereby a unified understanding of steroidogenesis in reproductive organs remains enigmatic.

Despite the promising implications of autophagy (as summarized in Table 1) in preclinical investigations, and as of our current knowledge, there exists a notable literature gap and further elucidation of these mechanistic insights is imperative to advance our comprehension and potentially pave the way for novel therapeutic interventions in reproductive health. Therefore, we, herein, explore the involvement of autophagy in ovarian and testicular steroidogenesis, along with the scrutiny of its regulatory mechanisms.

**TABLE 1:** Summary of recent studies investigating autophagy in testis and ovaries.

Sr	Author	Year	Methods	Major findings	Implications for reproductive health	Ref
1	Esmaeilian et al.	2023	Pharmacological inhibition, genetic interruption via siRNA and shRNA, and *Ex vivo* explants of testicular and ovarian tissue	Lipophagy is crucial in sex hormone production GTH upregulates ATGs and accelerates autophagy via promoting its link with autophagy	Potential treatments for reproductive disorders and related neoplasms	Esmaeilian et al. (2023)
2	Aldawood et al.	2020	*In vivo*, treatment with acrylamide*,* histological, hormonal, TUNEL analyses, and real-time-PCR	acrylamide reduces ovarian demographics and estradiol concentrations. Pyknotic characteristics and nuclear fragmentation. Apoptosis of granulosa cells. High doses induced overexpression of various autophagy genes	potential reproductive toxicity of acrylamide that warrants further exploration	Aldawood et al. (2020)
3	Harrath et al	2022	Inhalation exposure of female rats to ethylbenzene at various doses for 30 min daily for 1 month	Ethylbenzene disrupts ovarian function, leading to decreased growing follicles, increased abnormal follicles, accelerated reproductive aging, and imbalances in reproductive hormones	Ethylbenzene exposure adversely affects ovarian structure and function and triggers autophagy and apoptosis	Harrath et al. (2022)
4	Yong et al.	2021	Estrus cycle analysis, Immunohistochemistry, Immunofluorescence staining, and Western blotting	Malathion disrupts the estrus cycle, reduces ovarian hormone secretion, increases ROS, and induces ovarian autophagy and apoptosis; resveratrol mitigates these effects	Resveratrol is a potential preventive measure against malathion-induced ovarian damage and estrus cycle disorders	Yong et al. (2021)
5	Gao et al.	2018	Steroidogenic disruptions of autophagy in specific cells	Autophagy upregulates cholesterol intake in Leydig cells	Impaired autophagy contributes to declined testosterone in patients with hypogonadism	Gao et al. (2018a)
6	Long et al.	2022	Neonatal cryptorchid infertile rats, *in vivo* RA supplementation, *in vitro* testicle culture with RARα antagonist	RA upregulates c-Kit, Stra8, and Sycp3, and regulates PI3K-Akt-mTOR signaling, autophagy, and blood-testis barrier permeability in cryptorchid rats	RA treatment offers potential therapeutic strategies for cryptorchidism-related infertility	Long et al. (2022)
7	Rejani et al.	2022	Experimental dietary intervention on prepubertal rats	A prepubertal HFD-HF induces hypogonadotropism and autophagy-facilitated defective ovarian follicle differentiation, impacting the fertility of adult rats	Early exposure to an HFD-HF diet negatively affects gonadotropin levels and disrupts autophagy-mediated ovarian follicle differentiation, potentially influencing reproductive health in adulthood	Rejani et al. (2022)
8	Xie et al.	2021	Analysis of melatonin expression levels in PCOS patients and dehydroepiandrosterone -induced PCOS rat model	Reduced melatonin expression in PCOS patients, correlation with testosterone and cytokine levels, protective effect on ovarian function via PI3K-Akt pathway regulation	Melatonin is a potential target in the treatment of PCOS and improving ovarian function through autophagy modulation	Xie et al. (2021)
9	Zhang et al.	2023	Overexpression of CIRBP in YGCs cultured at 32°C for 6 and 12 h	CIRBP overexpression induces autophagy in YGCs, enhancing E2 and P4 in response to hypothermia	Autophagy is essential in the synthesis and secretion of ovarian hormones under mild hypothermic conditions	Zhang et al. (2023)
10	Chen et al.	2019	*In vivo* and *in vitro* exposure to AFB1 rat varying concentrations	AFB1 exposure reduced testosterone, LH, and FSH levels, quantitatively decreased Leydig cells and induced apoptosis via AMPK/mTOR-mediated autophagy	AFB1 adversely affects Leydig cell function, suggesting potential reproductive health risks related to endocrine disruption and cell apoptosis	Chen et al. (2019)
11	Meng et al.	2020	Perinatal exposure study with pregnant SD rats	Perinatal bisphenol A exposure advances puberty, increases E2, LH, and FSH concentrations, and affects endometrium thickness in female offspring	Adverse effects linked to impaired autophagy via TLR4/NF-κB and mTOR pathways	Meng et al. (2020)
12	Liu et al.	2020	*Ex vivo* experiments using rat ovarian granulosa cells treated with NP at various concentrations	NP exposure led to a significant reduction in granulosa cell viability, increased apoptosis with G2/M arrest, induction of autophagy, and elevated ROS production. Autophagy inhibition enhanced NP-induced apoptosis, and ROS inhibition with N-Acetyl-l-cysteine attenuated autophagy and cell death	NP exposure may adversely impact female reproductive health by promoting apoptosis and autophagy in ovarian granulosa cells through the activation of the ROS-dependent Akt/AMPK/mTOR pathway	Liu et al. (2020a)
13	Chen et al.	2022	Experimental exposure to nano-copper at varying doses for 28 days	Nano-copper impairs sperm quality, fructose, and hormone secretion, increases ROS, and alters testes	Nano-copper harms male reproduction via AKT/mTOR and oxidative stress	Chen et al. (2022a)

^a^ AFB1, aflatoxin B1; CIRBP, cold-induced RNA-binding protein; FSH, follicle stimulating hormone; HFD-HF, high-fat-high fructose diet; NP, nonylphenol, PCOS, polycystic ovary syndrome, RA: retinoic acid, ROS: reactive oxygen species.

## Autophagy regulation

**FIGURE 1:**
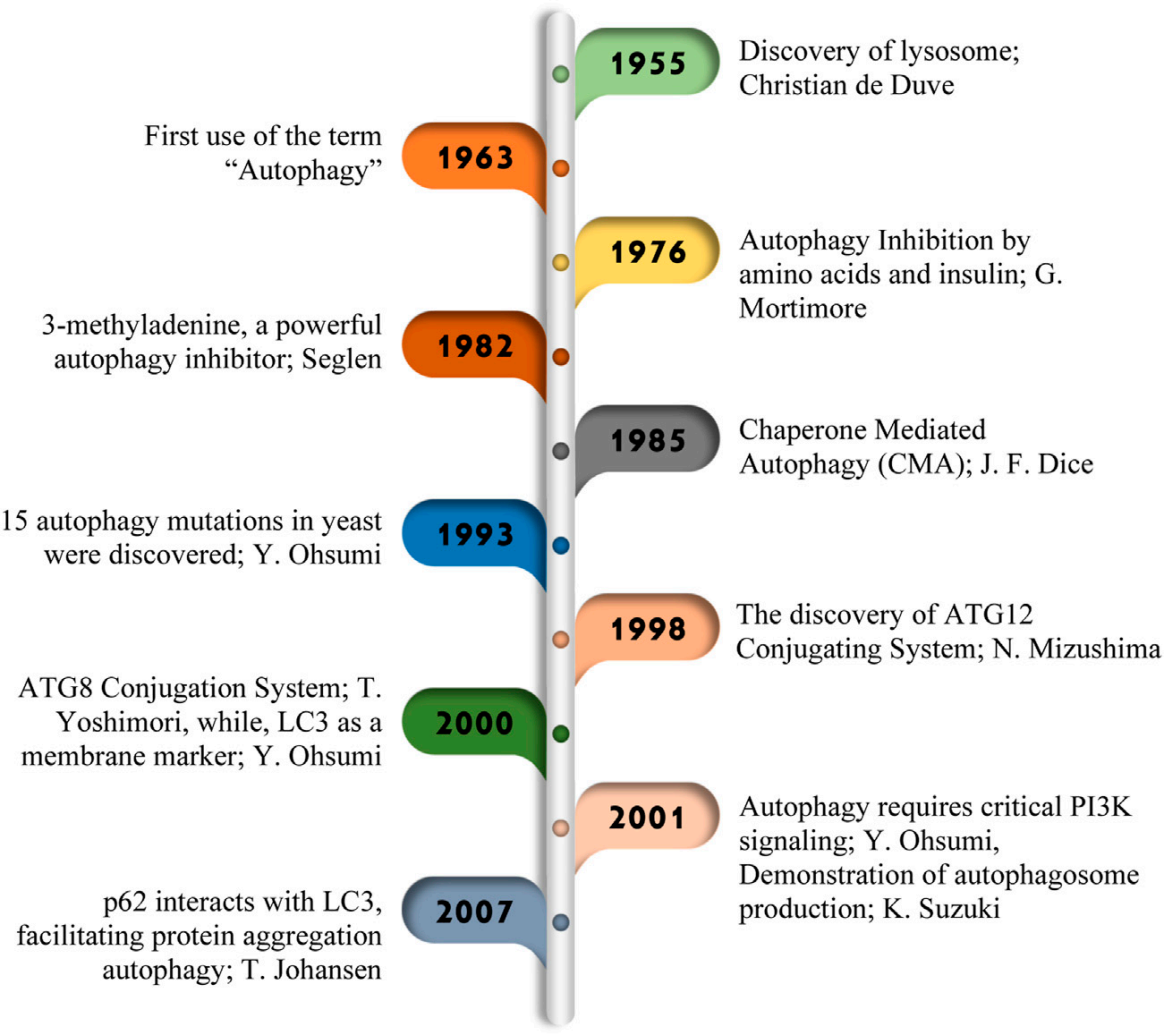
Major research discoveries in autophagy. In 1955, Christian de Duve coined the term “autophagy,” (Sabatini and Adesnik, 2013) paving the way for later discoveries of 1963 (Klionsky, 2008), G. Mortimore in 1976 (Mortimore et al., 1996), Seglen in 1982 (Gordon and Seglen, 1982), J. F. Dice in 1985 (Dice, 2007), Y. Ohsumi in 1993 (Tsukada and Ohsumi, 1993). Important breakthroughs in 1998 (Mizushima, 2020), including the identification of ATG genes (Sou et al., 2008), the ATG conjugation system (Choi et al., 2018), and insights into autophagosome formation (Shibutani and Yoshimori, 2014), marked significant progress in understanding this important cellular process.

**FIGURE 2:**
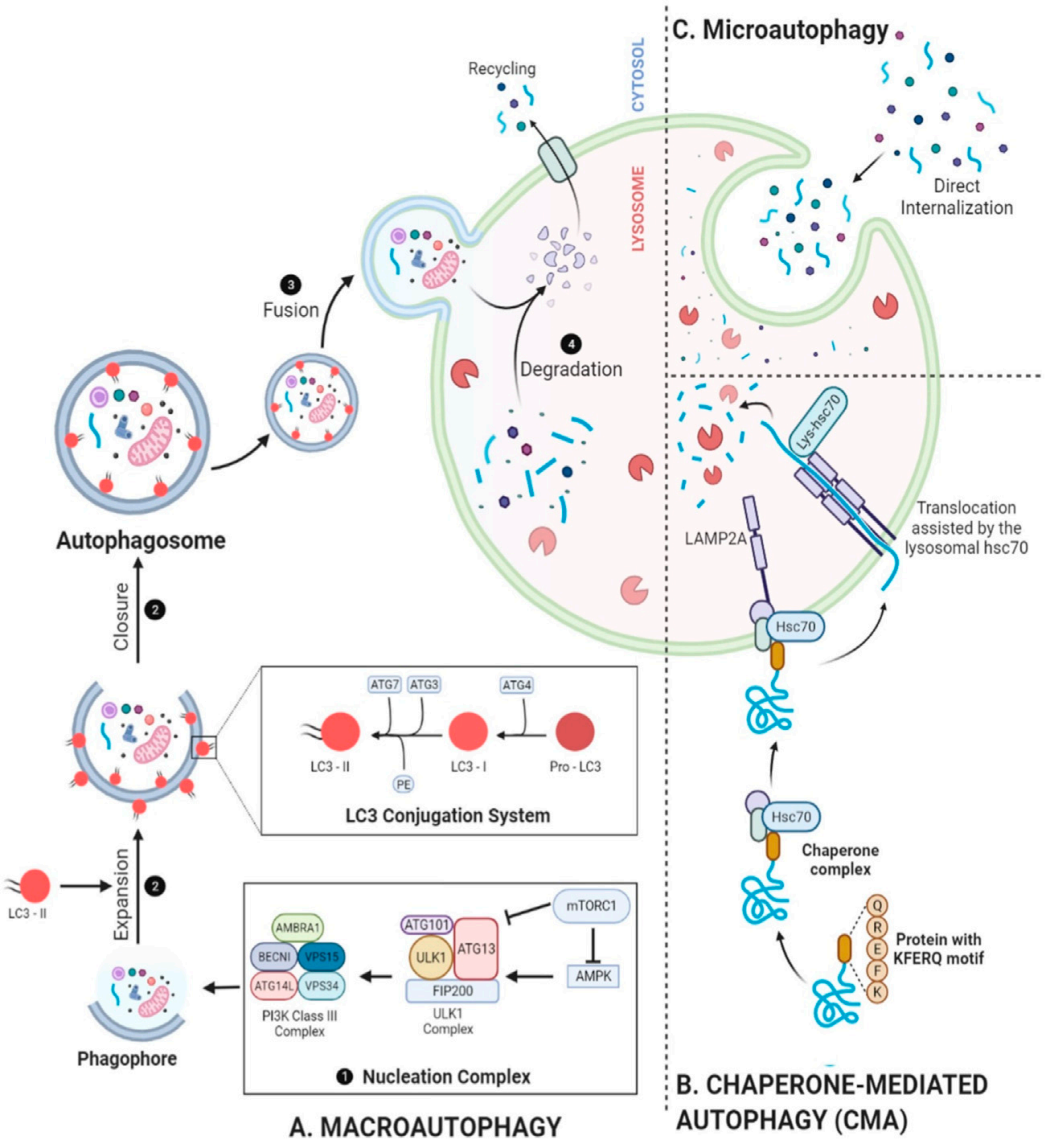
Mechanistic depiction of autophagy. **(A)** Beginning with nucleation of a phagophore, PI3K complex phosphorylation, and conversion of pro-LC3 into LC3-I and then into LC3-II through ATG4 processing, autophagy proceeds to conjugation with PE, facilitated by ATG3 and the ATG12–ATG5–ATG16L complex, results in the formation of LC3-II. This event promotes the expansion of the cup-shaped phagophore, leading to the formation of autophagosomes and the sequestration of cargo. Following fusion with a lysosome, the autophagosome exposes its cargo to lysosomal hydrolases, helping with degradation. **(B)** Microautophagy depicts the complete sequestration of components by lysosomes. **(C)** CMA selectively targets specific proteins for degradation by translocating them into lysosomes. These additional layers of autophagy further contribute to the complexity and specificity of cellular degradation processes, ensuring cellular homeostasis and adaptation to various physiological conditions.

Autophagy, a finely tuned and multi-step mechanism, is primarily regulated by autophagy-related (ATG) proteins that have remained evolutionarily conserved from yeast to mammals (Tsukada and Ohsumi, 1993; Yang and Klionsky, 2010). Figure 1 illustrates additional significant findings in autophagy research. Following initiation, various regulators come into play at the phagophore assembly site, guiding the progression through subsequent stages, including 1) nucleation, 2) expansion and closure of the phagophore, 3) maturation, and 4) degradation. Several genes regulate this mechanism (Figure 2) including the mechanistic target of rapamycin (mTOR), an inhibitory regulator (Ariosa et al., 2021) consisting of complexes; the mTOR complex I (mTORC-I) and mTORC-II of which the former responds to nutrient stress and later is influenced by growth factors or via PI3K/Akt signaling (Lim et al., 2021). Non-selective autophagy, also known as mTORC1-dependent autophagy, is triggered by nutrient stress and a low ATP/AMP ratio (Whitmarsh-Everiss and Laraia, 2021). ULK1 complex activation phosphorylates the ATG13 and FIP200 (Nie et al., 2021) which then leads to the phosphorylation of the PI3K class III complex I, starting phosphatidylinositol 3-phosphate (PI3P) production on the surface of omegasome–the assembly site of phagophore on the endoplasmic reticulum (ER) (Nishimura et al., 2017). PI3P recruits WIPIs and DFCP1 to the site. The expansion of the phagophore involves two complexes: The ATG12–ATG5–ATG16L1 complex and ATG8-family proteins like LC3-II, a product of the LC3 conjugation cascade, which facilitates phagophore expansion and closure, forming an autophagosome that sequestrates the cargo. Previously, we showed that SIRT1 is responsible for the deacetylation of LC3 within the nucleus from where it relocates to the cytoplasm and engages in the process of autophagosome formation via interacting with other autophagic components (Khawar et al., 2021b).

Selective or mTORC1-independent autophagy is mediated by a total of 26 mammalian receptors as summarized by Vargas et al. (2023). Following the phagophore closure, the subsequent steps involve the fusion of lysosome with autophagosome, giving rise to an amphisome—a non-degradative, single-membrane structure that further matures into an autolysosome (Zhao et al., 2021). Autophagosome acidification, facilitated by multi-subunit complexes comprising V1 and V0 sectors known as V-ATPase, is important for hydrolysis. Then LC3-II complexes are dissociated from the outer surface. A recruitment of machinery ensues, orchestrating the lysosomal fusion into autophagosomes which encompasses soluble N-ethylmaleimide sensitive fusion protein attachment protein receptors, various small GTPases, and their respective effector proteins (Lőrincz and Juhász, 2020). The Homotypic Fusion and Protein Sorting complex has been implicated in mediating the fusion between lysosomes and autophagosomes (Kondapuram et al., 2019). Following the maturation, sequestrated cargo undergoes cathepsins-mediated degradation. The resulting degraded material is transported back into the cytosol through lysosomal efflux transporters, e.g., the sugar transporter Spinster which is used for cellular building or energy production (Zhao et al., 2021). In case of prolonged starvation-induced autophagy, lysosomes undergo recycling through a process called autophagic lysosome reformation, regulated by an mTOR-dependent pathway. More comprehensive mechanistic insights into autophagy are available (Levine and Klionsky, 2004; Klionsky, 2005; Mizushima et al., 2008; Vargas et al., 2023). While our knowledge of the molecular entities in mammalian autophagy continues to expand and solidify, much remains to be elucidated. A comprehensive understanding of the regulatory mechanisms, encompassing both internal and external signals as well as downstream effectors, is essential. Such insights will significantly influence future therapeutic strategies for a variety of clinical disorders.

## Autophagy in testicular steroidogenesis

Testosterone, mainly produced in Leydig cells, portrays an essential function in the development of male characters and the sustenance of sexual function. Free cholesterol (FC) is a precursor for the biosynthesis of testosterone and can be obtained through various ways: 1) *de novo* production via acetic acid; 2) breakdown of accumulated esters of cholesterol, found in lipid droplets (LDs) or cell membranes, 3) extraction of serum lipoproteins, particularly high-density lipoprotein (HDL) (Thomas et al., 2012; Choi et al., 2021). Membrane carriers, e.g., the steroidogenic acute regulatory (StAR) protein, are important in relocating FCs to mitochondria (Galano et al., 2022). Inside the mitochondria, the enzyme cholesterol side chain cleavage (CYP11A1) converts FC into pregnenolone, which later, moves to the smooth ER, where it undergoes processing by three steroidogenic enzymes, ultimately leading to the synthesis of testosterone (Luetjens et al., 2012).

In rats, Leydig cells responsible for testosterone secretion show elevated autophagy levels compared to Sertoli cells (Xu et al., 2023) and primary Leydig cells (Zhang et al., 2022). Specifically, autophagosomes selectively target organelles important for steroid production, for example, mitochondria and smooth ER, within Leydig cells. This implies a potential role for autophagy in the steroid synthesis process. Moreover, inhibited cells experience an increase in autophagy levels, while luteinizing releasing factor-stimulated cells see a decrease; the autophagic activity level correlates with steroid release rates (Yi and Tang, 1995). This modulation pattern in autophagy resembles the observed crinophagy in pituitary cells which are responsible for peptide secretion. Since steroids are not stored in secretory bodies, Leydig cells managing excess secretory material degrade organelles involved in steroid production (Yi and Tang, 1995). Therefore, classical autophagy in rat Leydig cells seems to regulate steroid secretion, much like how crinophagy regulates peptide secretion in endocrine cells. It is likely that steroidogenic cells in the ovary and adrenal gland also engage in this process.

**FIGURE 3:**
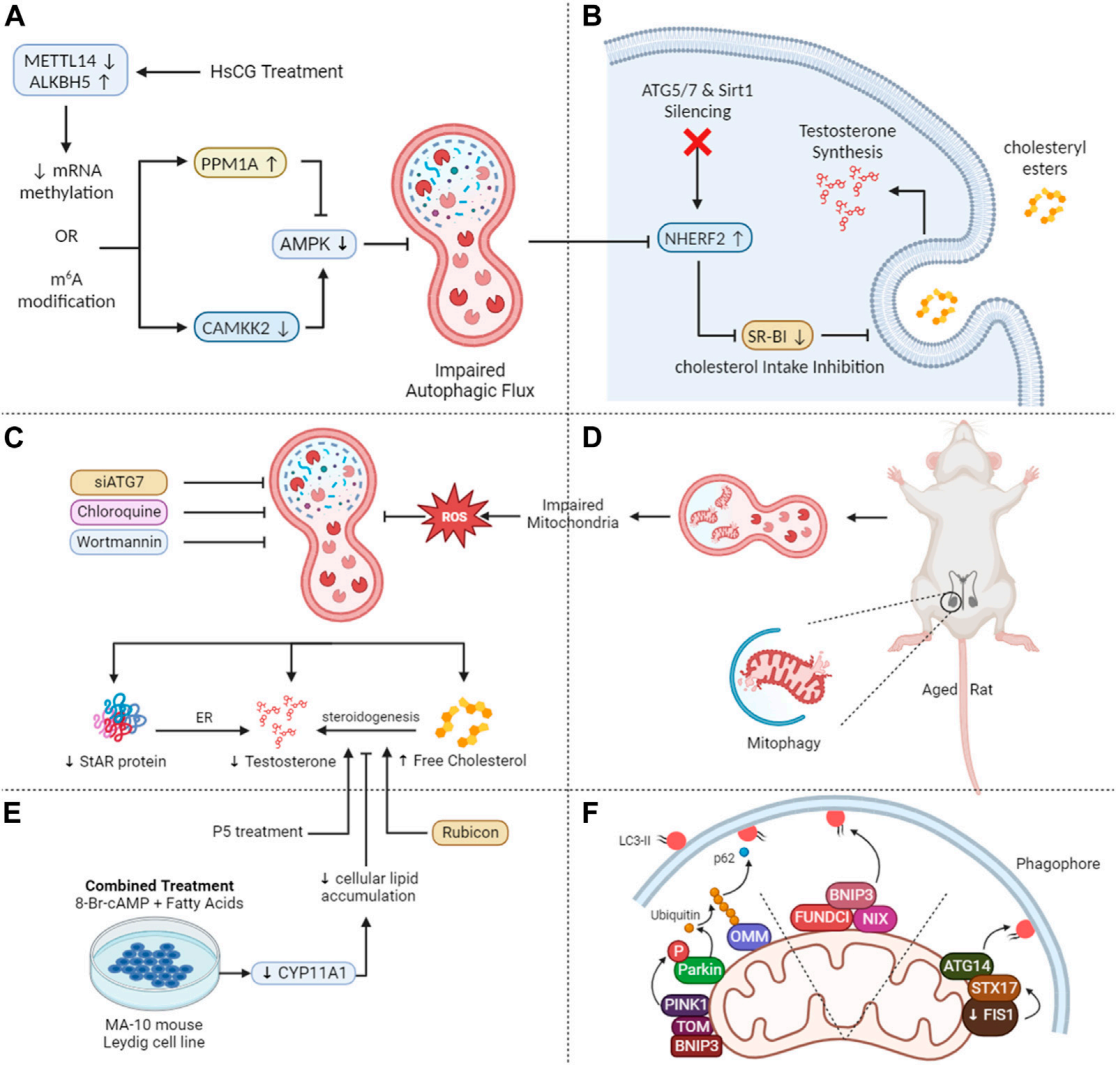
Mechanistic insights into autophagy-mediated steroidogenesis in testes. **(A)** HsCG causes a decrease in METTL14 while an increase in ALKBH5 leads to reduced N6-methyladenosine (m6A) levels via translation of PPM1A and CAMKK2 and reduced AMPK activity thus inhibiting autophagy. **(B)** The autophagy-lysosome pathway mediates the breakdown of NHERF2, suppressing scavenger receptor class B type I (SR-BI) and compromising cholesterol absorption and testosterone production when ATG5, ATG7, and Sirt1 are deleted in Leydig cells. **(C)** Autophagy inhibition in aged rat Leydig cells lead to decreased testosterone and free cholesterol levels and increased total cholesterol and lipid droplets. Stress reduces lipid droplets but increases testosterone release, effects counteracted by inhibiting autophagy. **(D)** Diminished mitophagy, in aging Leydig cells results in dysfunctional mitochondria accumulation, leading to ROS buildup, and reduced testosterone levels. **(E)** Fatty acid treatment in MA-10 cells leads to lipid accumulation and decreased steroidogenesis, recoverable by P5 but not 22R-OHC, indicating a deficiency in CYP11A1. Autophagy inhibition, including FA-upregulated Rubicon, suppresses steroidogenesis. **(F)** PINK1/parkin mitophagy: Parkin phosphorylation, outer mitochondrial membrane protein ubiquitination, phagophore recruitment. Non-parkin mitophagy: outer mitochondrial membrane proteins bind LC3 or STX17-mediated, with STX17 accumulating on outer mitochondrial membrane, interacting with LC3 on the phagophore membrane.

Notably, targeting the differentiation of Leydig cells and subsequent testosterone production via genetic manipulation in the form of N6-methyladenosine (m6A) modification is of great significance owing to various regulatory roles in spermatogenesis (Li et al., 2024), tumor development (Diao et al., 2023) and embryogenesis (Muhammad Babar et al., 2023). Therefore, its manipulation in the current scenario was recently accompanied by Chen et al. (2020) when they investigated m6A methylation in regulation of Leydig cell differentiation and their subsequent physiology. They underscored a pivotal function of m6A RNA methylation in regulating testosterone production in Leydig cells through autophagy modulation as shown in Figure 3A. This discovery opens up avenues for innovative therapeutic strategies targeting m6A RNA methylation to address reduced serum testosterone levels in patients with oligospermia and azoospermia (Chen et al., 2021).

Our recent research reveals that autophagy is pivotal in testosterone synthesis by supplying essential substrates. The scavenger receptor class B type I (SR-BI) aids the selective intake of cholesteryl esters having its source in lipoproteins. These findings indicate that the autophagy-lysosome pathway breaks down the Na^+^/H^+^ exchanger regulatory factor 2 (NHERF2), which functions as a suppressor of SR-BI. Consequently, the accumulation of NHERF2 leads to the suppression of SR-BI when ATG5, ATG7, and Sirt1 are deleted in murine Leydig cells, resulting in compromised cholesterol absorption and decreased testosterone production as depicted in Figure 3B (Gao et al., 2018a; Khawar et al., 2021b; Khawar et al., 2022).

Lipophagy plays a pivotal role in lipid metabolism in adipocytes, macrophages, and hepatocytes (Xie et al., 2020a; Xie et al., 2020b). In our recent review, we underscored the role of autophagosomes in the sequestration of LDs, a process that involves transporting these droplets to lysosomes for subsequent breakdown into free fatty acids. This mechanism not only helps with the recycling of cellular components but also contributes to the liberation of essential building blocks such as free fatty acids (Khawar et al., 2019; Khawar et al., 2021a). When Leydig cells are hormonally stimulated, their LDs produce FC, a critical substrate for testosterone synthesis (Shen et al., 2016). When autophagy is hindered in primary rat Leydig cells through chloroquine (CQ) or siATG7, testosterone and FC levels decline significantly. Concurrently, there is an increase in total cholesterol (TC) and LD levels in serum-free media. Additionally, brief exposure to hypoxia leads to a reduction in LD size and quantity but fosters an increase in testosterone release (Figure 3C). These effects can be counteracted by inhibiting autophagy (Ma et al., 2018). Hence, autophagy promotes testosterone production via metabolizing intracellular LDs and TC. Notably, lipophagy activity has been observed in the Chinese soft-shelled turtle as well (Tarique et al., 2019). Recently, within the Leydig cells of dairy goats, macroautophagy has been recognized as the predominant mechanism driving testosterone production. This is accomplished by breaking down mitochondria and ER (Chen et al., 2022b).

Notably, there may be additional connections between testosterone and autophagy. Deficiencies in autophagy have been observed to correlate with reduced testosterone levels and StAR protein in aged rat Leydig cells (Figures 3C, D). Moreover, these conditions are further characterized by higher levels of reactive oxygen species (ROS) and impaired mitochondria function. Leydig cells, regardless of age, showed reduced testosterone levels and LH-stimulated StAR when exposed to the autophagy inhibitor wortmannin. Conversely, the rapamycin–an autophagy promoter, produced contrasting outcomes in older Leydig cells. Considering that ROS impede StAR and testosterone production in Leydig cells, it is conceivable that the deposition of ROS in elderly rats might compromise Leydig cell steroidogenesis due to decreased autophagic activity (Han et al., 2018).

A notable decrease in testosterone synthesis in Leydig cells has been linked to a substantial drop in autophagic activity in non-breeding male natal naked mole rats. Treating primary Leydig cells from both breeding and non-breeding naked mole rats with rapamycin increased testosterone levels, whereas 3-MA had the opposite outcome (Yang et al., 2017). These observations reinforce the view that autophagy regulates testosterone biosynthesis. Consequently, we posit that individuals with reproductive concerns, particularly those diagnosed with varicocele or hypogonadism, should exercise caution when considering autophagy-inhibiting medications such as rapamycin. Notably, CQ, a drug commonly prescribed for early malaria treatment and prevention, is also used for conditions like rheumatoid arthritis and lupus erythematosus (Rainsford et al., 2015). As an autophagy inhibitor, CQ might adversely impact male fertility by suppressing autophagy, thereby potentially disrupting male steroid homeostasis and compromising testicular structural integrity (Clewell et al., 2009).

Recent studies have associated autophagic insufficiency with a decline in testosterone synthesis within aged rat Leydig cells. There is ample evidence documenting age-related reductions in autophagy (Mizushima et al., 2008; Cuervo et al., 2017). Diminished autophagy, especially mitophagy—the mTORC1-independent breakdown of damaged mitochondria—leads to a reduced removal of dysfunctional mitochondria in aging Leydig cells resulting in the accumulation of ROS which ultimately reduce the testosterone levels (as illustrated in Figure 3F), thereby contributing to conditions like late-onset hypogonadism (Li et al., 2011). This fact owes to ROS that detrimentally affects Leydig cell steroidogenesis (Zirkin and Chen, 2000; Diemer et al., 2003). Conversely, emerging research indicates that while autophagy enhances steroidogenesis and augments the steroidogenic efficacy of Leydig cells, it may not represent the primary biological mechanism governing steroidogenesis, especially in mature Leydig cells (Park et al., 2023).

Particularly, in mitophagy (Figure 3F), VDAC1 serves as a key link between cytosolic proteins and mitochondrial contact sites (Papadopoulos and Zirkin, 2021). It forms a complex with the adenine nucleotide translocase protein, facilitating molecule movement across mitochondrial membranes (Garza and Papadopoulos, 2023). These proteins play a vital role in maintaining mitochondrial membrane integrity, crucial for regulating mitophagy (Lin et al., 2024). Disruption in their function or interaction can impair mitophagy, leading to cellular stress and dysfunction, potentially affecting overall autophagic processes.

Mitochondrial contact sites with the ER are vital for regulating ROS in gonadal cells. These specialized contact sites, termed mitochondria-associated membranes (MAMs), facilitate the exchange of lipids and calcium ions between the ER and mitochondria (Katti et al., 2023), crucial for maintaining cellular redox balance. Perturbations in MAM function can result in the accumulation of ROS (Fang et al., 2023), which impact both signaling pathways and cellular health. Notably, the ER-resident chaperone Sig-1R, concentrated at MAMs, governs calcium dynamics and responses to oxidative stress (Gottschalk et al., 2022), suggesting its potential as a therapeutic target for conditions linked to ROS-induced damage in Leydig cells. Moreover, under conditions of ER stress, MAMs exhibit heightened stability and prolonged duration, bolstering ATP production by mitochondria and ensuring an adequate energy supply to the ER (Gottschalk et al., 2022). This adaptive response aids in mitigating ER stress and reinforces ATP provision to support proper protein folding, highlighting the intricate interplay between MAMs, ROS regulation, and cellular homeostasis in gonadal cells.

Moreover, the interaction between mitochondria and LDs plays a crucial role in regulating steroidogenesis in gonadal cells. These contact sites facilitate the transfer of cholesterol into mitochondria (Guyard et al., 2022). Proteins like translocator protein (TSPO) and StAR are involved in mediating this transfer (Yue et al., 2023). Mutations in StAR can lead to lipoid congenital adrenal hyperplasia (lipoid CAH), affecting steroid production (Martín et al., 2021). Additionally, these contact sites influence the density and size of LDs, impacting cell function (Galano et al., 2021; Knight et al., 2022). Future research could explore how alterations in mitochondrial-lipid droplet interactions affect steroidogenesis and potential therapeutic interventions for reproductive disorders.

Androgen binding protein (ABP), a glycoprotein produced from Sertoli cells, expediates the transport of testosterone into the epididymis (Wistuba et al., 2023). In rats, follicle stimulating hormone (FSH) and testosterone synergistically boost the entry of ABP into the testicular tubules. Nevertheless, following FSH delivery, only a fraction of ABP in the testis showed an increase, implying that testosterone likely serves as the primary regulator of ABP synthesis *in vivo* (Danzo et al., 1990). Detailed investigations in rat Sertoli cells suggest that testosterone specifically modulates the autophagy-mediated lysis of ABP *in vitro* and *in vivo*. The very fact is supported through evidence showing that treatments with CQ or rapamycin had no impact on ABP expression after testosterone exposure. Additionally, the hypothesis that testosterone acts as a pivotal regulator in the autophagic degradation of ABP gains credence from observations that ABP clearance remains unaffected by stress-induced (hypoxia) autophagy (Ma et al., 2015).

Autophagy facilitates the production of testosterone through the provision of resources (Gao et al., 2018b; Khawar et al., 2021b). However, it is noteworthy that testosterone exerts an inhibitory effect on autophagy (Ma et al., 2015). To maintain cellular homeostasis, The hypothesis proposes that testosterone operates in an inhibitory feedback manner on autophagy. This autophagic process could indirectly influence spermatogenesis by participating in the metabolic pathways associated with ABP and testosterone. Additionally, indications point to the existence of autophagy in the Sertoli cells of rat testes. In particular, rats subjected to ethanol display increased mitochondria-mediated germ cell death (Eid et al., 2002). The existence of autophagy in the Sertoli cells of these ethanol-exposed rats was corroborated through electron microscopy and immunohistochemical analyses of various ATGs, including LC3. Notably, a significant observation was the elevated occurrence of mitophagy. This suggests a potential anti-apoptotic function of mitophagy as compared to the ethanol-induced toxicity in Sertoli cells via eliminating impaired mitochondria and inhibiting the secretion of pro-apoptotic proteins (Eid et al., 2012).

Lipophagy enables the interaction between LDs and lysosomes via transporting lipids from LDs to lysosomes for degradation, resulting in the liberation of FC. Gonadotropin hormones not only upregulate autophagy-related genes but also expedite the autophagic flux. Additionally, they enhance the link between LDs, autophagosomes, and lysosomes, potentially leading to an augmented synthesis of gonadal steroids (Gawriluk et al., 2011). Moreover, luteinized granulosa cells (GCs) have been seen showing anomalies in women who exhibit impaired lutealization of ovaries. Specifically, these deviations occur at various stages of lipophagy-mediated progesterone (P4) synthesis. Such patients show reduced P4 synthesis, significant defects in the fusion of LDs with lysosomes, and a hindered progression of autophagy (Esmaeilian et al., 2023). Fatty acids reduced steroidogenesis in MA-10 cells via CYP11A1. P5, not 22R-OHC, partially restored it. Inhibiting late-stage autophagy, including FA-induced Rubicon, hindered steroidogenesis as shown in Figure 3E. Rubicon played a unique role, independent of late-stage autophagy inhibitors (Huang et al., 2021).

In addition, there is a recent surge in investigations that endocrine disrupting chemicals (EDCs) have been shown to influence steroidogenesis, and emerging evidence suggests potential effects on autophagy within steroid-producing cells (Zhu et al., 2019; Lahimer et al., 2023). For instance, when male Sprague-Dawley rats were exposed to high levels of bisphenol AF, there was a decrease in number of Leydig cells and a downregulation of CYP17A1, along with other genetic markers in Leydig and Sertoli cells. Specifically, bisphenol AF triggered autophagy by increasing levels of autophagy proteins like LC3B, Beclin1, and Bcl-2-associated X protein. This might have occurred through decreased phosphorylation of AKT1 and mTOR, ultimately leading to a reduction in steroid production in Leydig cells (Yu et al., 2022). Another EDC, methyl tert-butyl ether, similar to bisphenol AF, hinders the proliferation of Leydig cells, leading to apoptosis and mitophagy in the testes which was achieved by suppressing the phosphorylation of mTOR while promoting the phosphorylation of AMPK (Zhu et al., 2022). Most importantly, ketoconazole has also shown to inhibit steroidogenic enzymes which affects the steroidogenesis in gonadal cells (Gal and Orly, 2014). Additionally, Ketoconazole suppresses steroidogenesis via inhibiting CYP17A1, potentially through upregulation of miRNAs such as mir-22-5p, miR-671, miR-28-3p, and miR-92b-3p, which target CYP17A1 expression (Baitang et al., 2023). However, it is unclear how other EDCs such as, diethylstilbestrol would interact with ketoconazole in terms of pregnenolone and progesterone synthesis. Further research is needed to determine the potential interactions between ketoconazole and other EDCs in the context of steroidogenesis in testis.

These findings, in conjunction with prior research, hold substantial clinical implications. They pave the way for novel approaches to diagnose and treat a spectrum of conditions. This includes benign ailments like endometriosis and sex steroid-producing neoplasms, as well as malignancies reliant on sex steroids such as those affecting the prostate, breast, and endometrium. Additionally, understanding the interplay between EDC exposure, autophagy modulation, and steroidogenesis is crucial for elucidating the comprehensive mechanisms underlying gonadal function. While existing research underscores diverse roles for autophagy in the reproductive systems of humans and animals, a notable dearth of human data implies that much remains to be elucidated.

## Autophagy in ovarian steroidogenesis

**FIGURE 4:**
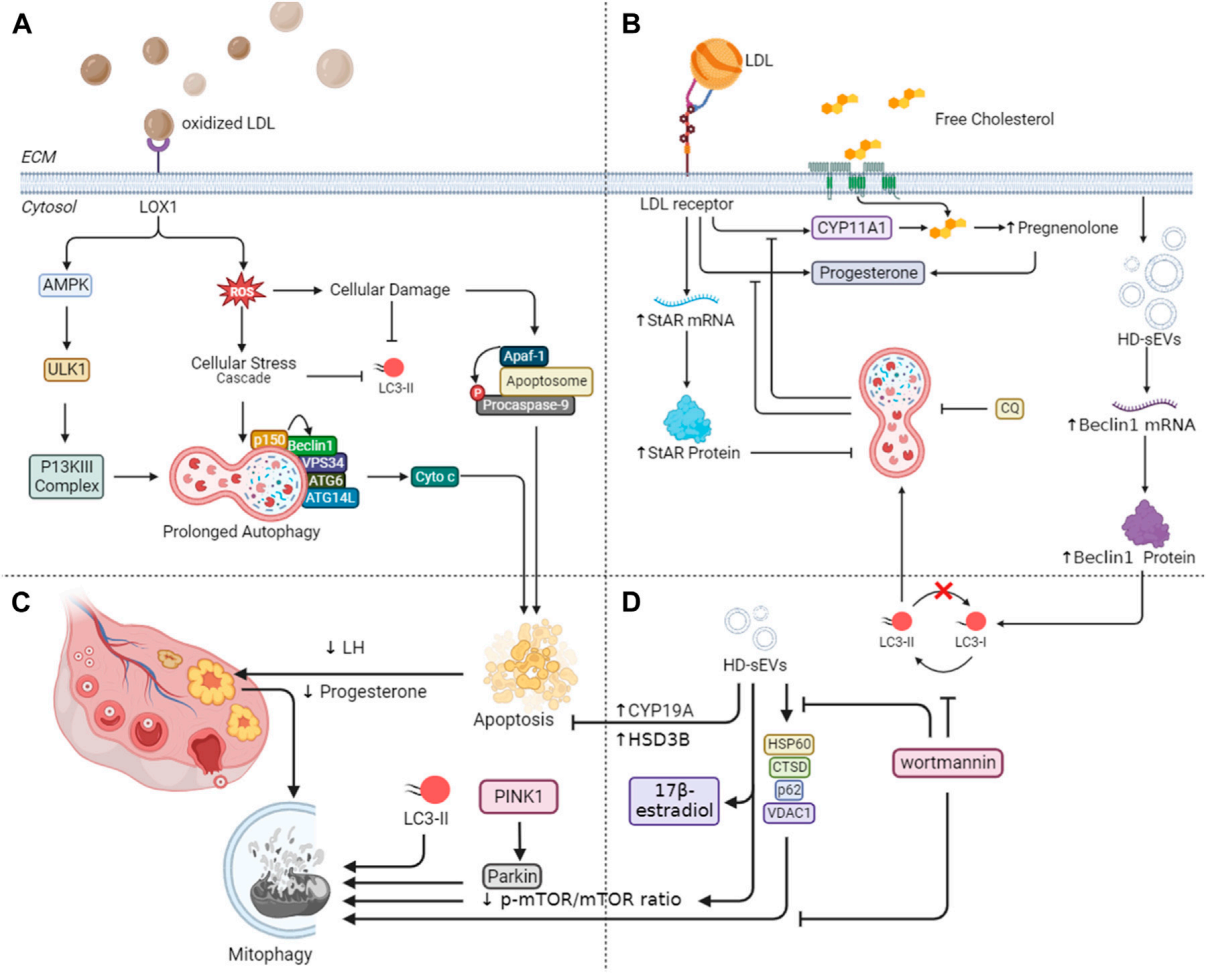
Mechanistic insights into autophagy-mediated steroidogenesis in ovaries. **(A)** Lectin-type oxLDL receptor (LOX1)-dependent oxidized LDL (oxLDL) in granulosa cell death through autophagy emphasizes its connection to obesity-related infertility. Additionally, it explores the age-associated decline in reparative autophagy, aligning with increased ROS levels and reduced LC3-II in older women. **(B)** Mitophagy-related proteins during luteal deterioration and highlights the impact of LDL on luteal development *in vitro*. **(C)** Beclin-1 expression and autophagy in granulosa cell longevity, especially in conditions of prolonged corpus luteum existence. **(D)** HD-sEVs from bovine follicular fluid on autophagy and mitophagy in bovine granulosa cells emphasize their role in reducing apoptosis and enhancing estradiol secretion through PI3K/Akt/mTOR signaling pathways.

Across various animal species, from *Drosophila* to mammals, autophagy is highly conserved being a critical player in the physiology of ovaries. In female physiology, autophagy emerges as a critical factor governing ovarian function. Specifically, critical for the survival of germ cells in development is autophagy, occurring before the development of the primordial follicle pool (Gawriluk et al., 2011). Initially, follicle atresia was attributed solely to apoptosis, however, recent discoveries suggest a more nuanced mechanism. For example, evidence indicates that human granulosa cell death is orchestrated through lectin-type ox-low density lipid (LDL) receptor (LOX1)-dependent oxidized LDL (oxLDL)-induced autophagy, suggesting the involvement of autophagic pathways in programmed cell death as shown in Figure 4A (Duerrschmidt et al., 2006; Serke et al., 2009). This finding potentially elucidates the heightened infertility rates observed in obese women, who exhibit elevated oxLDL levels (Mutlu-Türkoğlu et al., 2003) and an increased prevalence of autophagic granulosa cell death (Duerrschmidt et al., 2006).

Furthermore, elevated levels of ROS, resulting from LOX1 activation by oxLDL, can induce oxidative stress-induced apoptosis. Conversely, younger women with a normal weight seem to activate reparative autophagy as a response to reduced concentrations of ROS, thereby alleviating apoptosis, thus, supporting cell survival (Vilser et al., 2010). However, research by Tatone et al. (2006) and Vilser et al. (2010) suggests an age-associated decline in reparative autophagy, culminating in granulosa cell apoptosis. This decline aligns with observed increases in ROS levels and reductions in LC3-II in follicular cells from older women Figure 4A. Together, these discoveries align with the extensively recorded decrease in female fertility linked to the aging process (Carbone et al., 2003; Tatone et al., 2006; Tatone et al., 2008).

In animal models, these follicular cells have shown to undergo autophagy-mediated apoptosis in response to several stimuli, including nutritional scarcity and, more recently, exposure to cigarette smoke. These results suggest a plausible clarification for the established link between female infertility and smoking (Gannon et al., 2013). Following ovulation, the ovary produces the corpus luteum (CL). If conception does not take place, the CL regresses by the conclusion of menstruation. Electron microscopy studies have shown the accumulation of autophagy-related bodies, e.g., autophagosomes, in various cells during CL degeneration (Del Canto et al., 2007). At a molecular level, the human ovary possesses a specific voltage-gated Na channel that, when activated, starts downstream signaling leading to autophagy during CL regression (Bulling et al., 2000). Conversely, higher levels of Beclin-1 expression have been observed in pregnant CL cells and under pathological conditions where the CL persists longer than usual, suggesting a role for autophagy in granulosa cell longevity (Gaytán et al., 2008). Thus, we can conclude that autophagy has a dual role in regulating CL dynamics, as shown by a comprehensive review of both human and animal studies (Gao et al., 2020), highlighting its significance in ovarian function.

Recent studies indicate that the Akt/mTOR signaling pathway regulates the activation of ATGs in the CL. Furthermore, autophagy appears to influence progesterone synthesis by modulating the lipid droplet pool in luteal cells during pregnancy in rats. Notably, as luteal deterioration commences, there is an upregulation of mitophagy-related proteins, potentially essential for maintaining mitochondrial homeostasis (Figure 4C). These insights contribute to a more comprehensive grasp of the function of autophagy in the luteal changes of mammalian ovary *in vivo* in Sprague-Dawley rats (Tang et al., 2019). Moreover, in cultured bGCs, previous trials have shown that LDL upregulates StAR mRNA and protein (Figure 4B). Additionally, it promotes the production of progesterone and cholesterol side chain cleavage cytochrome P-450 (CYP11A1) mRNA. Furthermore, LDL considerably increases the lysosome count in GCs. However, the inhibitors of lysosomes, such as CQ effectively mitigate these effects induced by LDLs. These findings signify that in bGCs, LDL stimulates StAR expression, progesterone production, and lysosome development while lysosomes facilitate this process via secreting FC molecules from the breakdown of LDL (Zhang et al., 2015).

Recently, density gradient ultracentrifugation successfully isolated HD-sEVs from bovine follicular fluid (BFF) (Wang et al., 2023). These HD-sEVs induce autophagy in bovine bGCs by upregulating Beclin1 mRNA and protein expression, as well as increasing the LC3II/LC3I ratio (Figures 4B, D). Conversely, they suppress p62 mRNA and protein expression. HD-sEVs elevate the protein and mRNA levels of VDAC1, CTSD, and HSP60, subsequently promoting mitophagy in bGCs (as shown in Figure 4D). Flow cytometry results indicate that HD-sEVs diminish bGC apoptosis rates by upregulating steroidogenic proteins and mRNAs, including CYP19A and HSD3B in bGCs, HD-sEVs stimulate estradiol secretion. Additionally, HD-sEVs decrease the p-mTOR/mTOR ratio and boost autophagosome production and mitochondrial structural alterations in bGCs (Wang et al., 2023). The introduction of wortmannin reverses these observed effects. Mutually, BFF HD-sEVs enhance macroautophagy and mitophagy in bGCs, inhibit apoptosis in bGCs, and elevate 17β-estradiol release via the PI3K/Akt/mTOR signaling pathways as shown in Figure 4D (Wang et al., 2023).

Pups of *Mus musculus* with *ATG5*-knockout ovaries showed normal follicular development but lacked CL, displaying elevated atretic follicles, showing the unsuccessful release of an egg. Additionally, these pups showed a compromised ability of the uterus to produce the endometrial gland (Yoshii et al., 2016). These observations imply that autophagy is a vital player in the proper sexual development. To generate mice with ovarian-specific conditional knockout (cKO), *Beclin1*-knockout mice were employed, where Beclin1 was selectively deleted in granulosa and luteal cells (Gawriluk et al., 2014). This mouse model showed a nearly 75% reduction in Becn1 levels, with p62 accumulation observed in GCs. Although ovulation, fertilization, and implantation appeared similar to controls concerning reproductive phenotypes, the targeted elimination of Beclin1 led to a rise in miscarriages or premature births attributable to the failure of mitochondria to produce progesterone. Pathologically, ovaries with *Becn1*-knockout lacked neutral LDs responsible for progesterone production in luteal cells. Notably, these luteal cells exhibited many large autophagosomes, suggesting compromised autophagy processes, despite Beclin1’s role in nucleation—the initial phase of autophagy. The exact mechanism through which Becn1 downregulation increases autophagosome quantities in luteal cells remains unclear. In contrast, the overexpression of BECN1 in cultured GCs stimulated progesterone synthesis by boosting the production of synthesizing enzymes, including CYP11A1, 3β-hydroxysteroid dehydrogenase, and StAR protein (Ding et al., 2021). Recent research also suggests that FSH, regardless of the traditional steroidogenic pathway, enhances autophagy by upregulating Beclin1 through the PI3K/JNK/c-Jun pathway, promoting LDs breakdown in pig GCs (Liu et al., 2021). Furthermore, emerging evidence highlights the significance of autophagy in melatonin-mediated regulation of progesterone release in the sheep CL (Duan et al., 2024).

FSH from pituitary facilitates the progression of primary to dominant preovulatory follicles (Yoshino et al., 2011). Additionally, FSH causes the breakdown of LDs in porcine GCs (Liu et al., 2021), leading to the synthesis of progesterone via the Beclin1 protein. When porcine GCs were exposed to ATG5siRNA, promoting autophagosome formation, or CQ, an autophagy inhibitor, there was a notable decrease in FSH-induced progesterone production. This indicates the critical role of autophagy in progesterone synthesis within ovaries. Recent findings (Shao et al., 2022) underscore the importance of the gene regulator WT1 in follicle formation. Overexpression of WT1 affects normal granulosa cell development, while heterozygous mutations in WT1 result in subfertility in female mice, accompanied by reduced expressions of the FSH receptor and cytochrome P450 family 19 subfamily A member 1, commonly referred to as aromatase (Gao et al., 2014). Inhibiting autophagy leashes to a buildup of WT1 protein in GCs, diminishing the levels of receptor and protein, thereby disrupting GC differentiation (Galano et al., 2022). To modulate WT1 levels, Epg5 facilitates WT1 breakdown via p62 in GCs. Mice with Epg5 deletions display a phenotype akin to individuals with premature ovarian failure (Liu et al., 2023). Aging is linked with increased apoptosis and senescent cells. In the absence of Epg5, GCs in the ovary retain WT1, which would typically be degraded during folliculogenesis from secondary to antral follicles, resulting in subfertility in mice. Conversely, during folliculogenesis—where differentiation is not reliant on FSH—bone morphogenetic protein-2 (BMP2) improves GC proliferation through sphingosine kinase-1 (Ito et al., 2021). Considering that WT1 modulates the expression of BMG2 (Gao et al., 2014), it would be plausible that autophagy influences the entire spectrum of folliculogenesis in the ovaries, from early to late stages.

As it is a fact that steroidogenesis is a complex process which involves several enzymes and regulatory factors with pivotal roles from cholesterol transport to the synthesis of specific hormones within different cell types (Shoorei et al., 2023), thereby making it crucial for the production of key sex steroids. The interplay between hormones, receptors, and enzymes like CYP450arom highlights the intricate regulatory mechanisms governing steroidogenesis (De Pascali et al., 2018; Widhiantara et al., 2021). Several studies have reported variations in sex hormone synthesis upon bisphenol A exposure. For instance, when rat offsprings prenatally exposed to bisphenol A it disrupted steroid production (Nguyen et al., 2020). Higher bisphenol A doses of 40 mg kg^−1^ made the pregnant rats vulnerable to abortion and significant reduction of pups survival (Wei et al., 2020). A study on freshwater fish, *Gobiocypris rarus,* showed DNA and histone methylation in the ovaries and long term and short term exposure lead reduced steroid hormones (Liu Y. et al., 2020).

In short, autophagy is vital for ovarian physiology, influencing folliculogenesis, ovulation, and luteal development, impacting germ cell survival and fertility. Age-related decline correlates with increased oxidative stress and reduced fertility in older women, while autophagy plays a dual role in CL dynamics and modulates progesterone synthesis. Additionally, understanding the impact of endocrine disruptors on autophagy-mediated steroidogenesis in ovarian cells is essential for comprehending their broader effects on hormonal balance. Genomic studies underscore its significance in sexual and follicular development, offering insights into reproductive health.

## Conclusion and perspectives

The complex interplay between autophagy and steroidogenesis, particularly in the endocrine system, reveals a fascinating landscape of cellular mechanisms and pathways. Autophagy, a fundamental cellular process, has been recognized as a critical player in maintaining cellular homeostasis, eliminating damaged components, and facilitating various physiological functions. Its role in the context of steroid hormone production, especially testosterone and progesterone, underscores its significance in reproductive health and endocrine regulation. The research elucidates the nuanced role of autophagy in Leydig and GCs, pivotal players in testicular and ovarian steroidogenesis, respectively.

From the degradation of organelles essential for hormone production to modulating the expression of crucial proteins involved in steroid synthesis, autophagy emerges as an important regulator. The autophagy modulation, as observed in various studies, directly correlates with changes in steroid production rates, indicating a tightly regulated relationship. Furthermore, insights into the regulatory mechanisms of autophagy, such as the involvement of specific genes, signaling pathways, and external factors, provide a comprehensive view of its orchestration within the endocrine system. The intricate balance between autophagy and steroidogenesis has profound implications for understanding fertility, aging-related endocrine disorders, and potential therapeutic interventions. Given the significant impact of autophagy modulation on steroid production, future research may focus on developing targeted therapeutic interventions. Drugs that selectively enhance or inhibit autophagy in specific cells could offer novel treatments for conditions like hypogonadism, infertility, or age-related endocrine disorders. In addition, delving deeper into the molecular mechanisms underlying the interplay between autophagy and steroidogenesis will be important. Identifying additional genes, pathways, or molecules that regulate this relationship could provide more precise targets for therapeutic interventions.

While some studies have explored the effects of EDCs like bisphenol AF and methyl tert-butyl ether on Leydig cell function and hormone production, there’s still much to learn about how they affect different types of cells and hormonal pathways. Understanding how EDCs affect hormone production could help us identify ways to protect reproductive health. It is also crucial to understand the underlying molecular mechanisms behind changes in hormone levels seen with exposure to EDCs like bisphenol A. This knowledge could lead to new ways to prevent or treat hormone-related health issues.

Extending research from animal models to human studies will be pivotal. Investigating the role of autophagy in human Leydig and GCs, especially in pathological conditions like endometriosis, polycystic ovary syndrome, or testicular disorders, will provide invaluable clinical insights. Furthermore, given the age-related decline in autophagy and its implications for endocrine health, further research into rejuvenating autophagic processes in aging cells or tissues could have profound implications for extending reproductive health and overall longevity. To sum up, the intricate relationship between autophagy and steroidogenesis within the endocrine system represents a burgeoning field of research with vast clinical implications. By unraveling the complexities of this relationship, scientists and clinicians alike stand poised to revolutionize treatments for a myriad of endocrine disorders, ultimately enhancing reproductive health and quality of life.

## Abbreviations

ABP, Androgen binding protein; CL, Corpus luteum; CMA, Chaperone-mediated autophagy; CQ, Chloroquine; EDC, Endocrine disrupting chemicals; ER, Endoplasmic reticulum; FC, Free cholesterol; FSH, Follicle stimulating hormone; GC, Granulosa cells; LD, Lipid droplets; LDL, Low density lipid; MAM, Mitochondria-associated membranes; ROS, Reactive oxygen species; StAR, Steroidogenic acute regulatory; TC, Total cholesterol.
